# Analysis of the correlation between the Systemic Inflammatory Response Index and the severity of coronary vasculopathy

**DOI:** 10.17305/bb.2024.10747

**Published:** 2024-12-01

**Authors:** Ting He, Yinhua Luo, Jingjing Wan, Ling Hou, Ke Su, Jinbo Zhao, Yuanhong Li

**Affiliations:** 1 Department of Central Hospital of Tujia and Miao Autonomous; Prefecture, Hubei University of Medicine, Shiyan, Hubei Province, China; 2 Department of Cardiology, Zhongnan Hospital, Wuhan University, Wuhan, Hubei Province, China; 3 Institute of Myocardial Injury and Repair, Wuhan University, Wuhan, Hubei Province, China; 4 Cardiovascular Disease Center, Central Hospital of Tujia and Miao; Autonomous Prefecture, Hubei University of Medicine, Enshi, Hubei Province, China

**Keywords:** Systemic Inflammatory Response Index (SIRI), coronary heart disease (CHD), Gensini score, severity of coronary artery disease

## Abstract

The Systemic Inflammatory Response Index (SIRI) has been found to significantly correlate with the severity of coronary artery stenosis in patients with coronary heart disease (CHD), suggesting its potential as a reliable predictive marker. This study aims to analyze the correlation between the SIRI and the severity of coronary artery stenosis in patients with CHD. It also aims to assess the predictive value of SIRI for the severity of coronary artery stenosis. A total of 2,990 patients who underwent coronary angiography were included in this study. The Gensini score was used to estimate the severity of coronary vascular lesions. The predictive ability of SIRI for CHD was evaluated using receiver operating characteristic (ROC) curves. Binary multivariate logistic regression analysis was used to predict the likelihood of CHD based on the SIRI. The results showed that people with higher SIRI were more likely to have CHD (*P* < 0.001). After controlling for other risk factors, the highest quartile had a significantly higher incidence of coronary artery disease compared to the lowest quartile (odds ratio [OR] = 2.25, 95% confidence interval [CI] 1.73–3.92, *P* < 0.001). Furthermore, the Gensini score was significantly higher in the fourth quartile group (T4) compared to the first (T1) and second (T2) quartile groups (*P* < 0.001). Additionally, the SIRI was significantly higher in the group with severe coronary artery lesions compared to the mild and moderate groups (*P* < 0.001). The SIRI also showed a higher predictive ability for the extent of coronary lesions under the ROC curve compared to other commonly used markers, including platelet-to-lymphocyte ratio (PLR), monocyte-to-lymphocyte ratio (MLR), and neutrophil-to-lymphocyte ratio (NLR) (*P* < 0.001). Therefore, the SIRI positively correlates with coronary artery stenosis in CHD patients, serving as an effective early screening marker for assessing stenosis severity.

## Introduction

Globally, cardiovascular diseases (CVDs), particularly coronary heart disease (CHD), remain a leading cause of sickness and death. Nowadays, the main methods for lowering the risk of cardiovascular disease are lifestyle modifications and controlling common risk factors, such as diabetes, high blood pressure, and high cholesterol [1]. Even with the growing focus on managing health and successfully reducing traditional risk factors, the frequency of cardiovascular events is still quite high. Chronic inflammation is a crucial factor that cannot be disregarded. Numerous clinical and experimental research have provided compelling evidence that vascular inflammation plays a critical role in the development of atherosclerosis (AS) and acute coronary syndrome (ACS) [2–5]. In clinical settings, inflammatory cells and their products are often used as indicators of inflammation [6]. Combining two or three blood indicators can better reflect the inflammatory state in the body and have a synergistic effect on the clinical prognosis of patients with cardiovascular disease as well as the prediction of the risk of acquiring cardiovascular disease [7–9].

A recently proposed marker of the systemic inflammatory response, the Systemic Inflammatory Response Index (SIRI) has the benefit of being widely accessible and reasonably priced. Qi et al. [10] first proposed the index in 2016. It was shown in cohort research that the SIRI index might represent both the level of systemic inflammation and the local immune response. The application of the SIRI index in cardiovascular disorders has grown in popularity as the significance of inflammation in these conditions has been increasingly highlighted. Compared to the previous single and two composite indices, SIRI combines three blood parameters for a more stable and comprehensive response to the systemic inflammatory response. Lymphocytes are an important component of the SIRI index, which is a good indicator of the immune status of the organism and provides a good response to the prognosis of many diseases. Low lymphocyte count is closely related to the poor prognosis of cardiovascular and cerebrovascular diseases [11]. Similarly, the current research on the SIRI index mainly focuses on the risk of cardiovascular and cerebrovascular diseases, all-cause mortality, and prognosis. Studies have shown that the SIRI index is positively correlated with the degree of coronary artery stenosis in elderly patients with ACS [12]. Further research is needed to determine if this correlation applies to a broader population of patients with CHD. Therefore, this retrospective study examines the relationship between the SIRI index and coronary artery vascular disease in patients with CHD. The study aims to identify new predictors for early assessment, prevention, and intervention of CHD, and to aid high-risk individuals in managing their health effectively.

## Materials and methods

### General information

**Figure 1.**
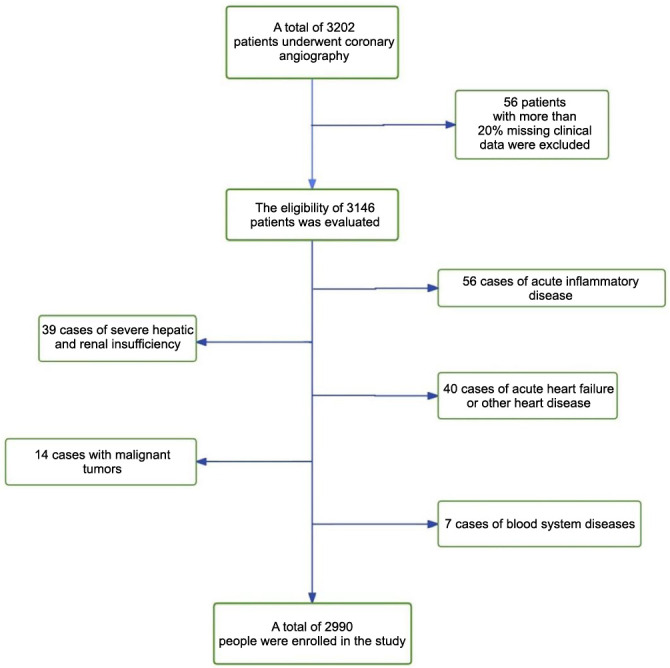
The selection procession of selected subjects.

This study investigated patients who were hospitalized and underwent coronary angiography in the Department of Cardiology at the Tujia-Miao Central Hospital of Enshi Autonomous Prefecture in 2023. The following were the exclusion criteria for this study: (1) cases with more than 20% missing data; (2) patients with congenital heart disease, heart failure, and other cardiac diseases; (3) patients with immune system disorders, malignant tumors, severe hepatic and renal insufficiency, and hematological disorders; (4) patients who have had acute inflammatory diseases, such as acute pneumonia, acute gastroenteritis, acute pancreatitis, and localized infections in the last month; and (5) patients with recent history of major surgical operation or medical history of cerebral hemorrhage or stroke; 2990 people who met the requirements were finally included in this study (Figure 1).

### Data collection

General demographic data of all patients were collected, such as age, gender, and smoking history; past medical history, mainly including history of hypertension, diabetes mellitus, and cerebrovascular disease (stroke, cerebral infarction); laboratory and clinical data. The first blood biochemical indices of the patients were collected after admission. Based on these laboratory results, the SIRI index of the patients was calculated [10] from peripheral blood neutrophil count (×10^9^/L) × monocyte count (×10^9^/L)/lymphocyte count (×10^9^/L). And the patients were divided into four groups according to the quartiles of the SIRI index; using the Gensini score [13] to assess the severity of coronary artery vasculopathy, and record the number of coronary artery vasculopathy of each patient.

### Ethical statement

This study was approved by the Ethics Committee of Enshi Autonomous Prefecture Central Hospital in accordance with the Helsinki Declaration, Decision No: 2024-052-01. All patient data used in this article is completely anonymous and the data has been anonymized prior to access and analysis; thus, there is no potential risk to individual patients or their personal privacy that would threaten it. As the committee did not require patient consent for the review of their medical records, obtaining informed consent from patients is not necessary for this retrospective study.

### Statistical analysis

SPSS 23.0 statistical software was used for data analysis. Continuous variables were expressed as mean ± standard deviation (SD) or median and interquartile spacing. For normally and non-normally distributed data, differences between groups were assessed using the independent samples *t*-test or Mann–Whitney *U* test, respectively. Categorical variables were expressed as numbers (percentages) and compared by chi-square test. When multivariate logistic regression was used to test the association of the SIRI index with different groups of coronary angiography data, the effect of confounders on the model was reduced by adjusting for age, sex, and other common risk factors. Subjects’ work characteristic curves (ROC) and area under the curve (AUC) were used to assess the ability of the SIRI index to predict AS. *P* values <0.05 were considered statistically significant. As this study was retrospective, it may have been affected by selection and recall bias.

**Table 1:** Clinical characteristics of individuals by SIRI quartile

**Characteristics**	**SIRI quartiles**				***P***
	**T1 (lowest) (*n* ═ 747)**	**T2 (*n* ═ 746)**	**T3 (*n* ═ 750)**	**T4 (highest) (*n* ═ 747)**	
Age (years)	59 (54, 68)	60 (53, 69)	61 (54, 70)^a^	64 (55, 72)^ab^	<0.001
SBP (mmHg)	130 (120, 146)	130 (120, 144)	130 (120, 148)	130 (120, 150)	0.813
HR (bpm)	72 (70, 78)	72 (70, 78)	72 (70, 79)	72 (70, 80)^abc^	<0.001
Male	281 (37.6%)	404 (54.2%)	526 (70.1%)	564 (75.5%)	<0.001
Smoking	194 (26.0%)	299 (40.1%)	377 (50.3%)	404 (54.1%)	<0.001
Hypertension	285 (38.2%)	322 (43.2%)	358 (47.7%)	347 (46.5%)	0.001
Diabetes	87 (11.6%)	113 (15.1%)	115 (15.3%)	148 (19.8%)	<0.001
WBC (10^9^/L)	4.86 (4.15, 5.63)	5.64 (4.84, 6.52)^a^	6.34 (5.46, 7.34)^ab^	8.57 (6.94, 10.51)^abc^	<0.001
NEUT (10^9^/L)	2.68 (2.23, 3.18)	3.48 (2.96, 4.02)^a^	4.23 (3.64, 4.96)^ab^	6.48 (5.05, 8.37)^abc^	<0.001
LYMPH (10^9^/L)	1.73 (1.40, 2.14)	1.63 (1.31, 2.02)^a^	1.46 (1.14, 1.81)^ab^	1.29 (0.96, 1.68)^abc^	<0.001
MONO (10^9^/L)	0.28 (0.23, 0.33)	0.35 (0.29, 0.41)^a^	0.39 (0.32, 0.46)^ab^	0.48 (0.38, 0.63^)abc^	<0.001
Hb (g/L)	137 (127, 147)	141 (130, 153)^a^	145 (132, 157)^ab^	146 (133, 157)^ab^	<0.001
PLT (10^9^/L)	193 (158, 226)	195 (161, 236)	198 (164, 235)^a^	202 (165, 238)^a^	0.003
ALT (U/L)	18 (13, 27)	20 (14, 28)	20 (15, 30)^a^	24 (16, 36)^abc^	<0.001
AST (U/L)	23 (19, 29)	23 (19, 29)	24 (19, 30)^a^	29 (21, 53)^abc^	<0.001
ALB (g/L)	40.61 (37.74, 43.44)	40.12 (37.39, 43.41)	40.41 (37.20, 43.51)	39.35 (36.34, 42.98)^abc^	<0.001
TC (mmol/L)	4.55 (3.81, 5.28)	4.39 (3.66, 5.12)	4.39 (3.58, 5.31)	4.39 (3.65, 5.27)	0.154
TG (mmol/L)	1.22 (0.88, 1.83)	1.31 (0.97, 1.92)	1.36 (0.97, 1.87)	1.38 (1.01, 2.12)^a^	<0.001
LDL-C (mmol/L)	2.74 (2.22, 3.26)	2.71 (2.12, 3.22)	2.71 (2.16, 3.33)	2.72 (2.18, 3.30)	0.599
HDL-C (mmol/L)	1.19 (0.99, 1.41)	1.15 (0.98, 1.34)^a^	1.09 (0.92, 1.29)^b^	1.08 (0.90, 1.28)^ab^	<0.001
ApoA1 (g/L)	1.40 (1.23, 1.65)	1.37 (1.21, 1.61)	1.33 (1.16, 1.57)^ab^	1.30 (1.15, 1.55)^ab^	<0.001
ApoB (g/L)	0.86 (0.67, 1.06)	0.86 (0.67, 1.06)	0.86 (0.69, 1.07)	0.87 (0.68, 1.10)	0.234
LPa (g/L)	0.102 (0.046, 0.262)	0.105 (0.049, 0.264)	0.119 (0.053, 0.271)	0.131 (0.053, 0.301)^a^	0.043
Cr (µmol/L)	61.9 (51.9, 74.4)	65.7 (55.9, 78.8)^a^	72.5 (60.9, 86.3)^ab^	75.3 (63.1, 89.8)^ab^	<0.001
GLU (mmol/L)	4.96 (4.45, 5.68)	5.05 (4.51, 5.96)	5.21 (4.61, 6.33)^a^	6.17 (5.11, 8.07)^abc^	<0.001
UA (µmol/L)	306.04 (257.03, 369.67)	326.30 (274.94, 392.95)^a^	345.67 (290.15, 407.75)^ab^	356.76 (294.89, 433.11)^ab^	<0.001

Data are given as median (IQR) or n (%). *P* < 0.001 were considered statistically significant; ^a^*P* < 0.001 vs T1; ^b^*P* < 0.001 vs T2; ^c^*P* < 0.001 vs T3. T1: The first SIRI index quartile; T4: The fourth SIRI index quartile; SBP: Systolic blood pressure; HR: Heart rate; WBC: White blood cell count; NEUT: Neutrophil count; LYMPH: Lymphocyte; MONO: Monocyte; Hb: Hemoglobin; PLT: Platelet count; ALT: Alanine aminotransferase; AST: Aspartate aminotransferase; ALB: Albumin; TC: Total cholesterol; TG: Triglyceride; LDL-C: Low-density lipoprotein; HDL-C: High-density lipoprotein; ApoA1: Apolipoprotein A1; ApoB: Apolipoprotein B; LPa: Lipoprotein a; Cr: Creatinine; GLU: Glucose; UA: Uric acid.

## Results

### Baseline characteristics

A total of 2990 patients who underwent coronary angiography were included in the study. The patients were divided into quartiles based on their SIRI index. Table 1 displays the demographics and characteristics of each group. The four groups of subjects did not show differences in SBP (systolic blood pressure), TC (total cholesterol), LDL-C (low-density lipoprotein), and ApoB (apolipoprotein B). However, the proportion of males, patients with hypertension, diabetes mellitus, and smokers was significantly higher in the highest quartile of the SIRI index (*P* < 0.001); white blood cell count (WBC), neutrophil count (NEUT), monocyte count (MONO), platelet count (PLT), alanine aminotransferase (ALT), aspartate aminotransferase (AST), triglyceride (TG), lipoprotein a (LPa), creatinine (Cr), glucose (GLU), and uric acid (UA) levels were elevated (*P* < 0.001); lymphocyte count (LYMPH), albumin (ALB), high-density lipoprotein (HDL-C), and apolipoprotein A1 (ApoA1) levels were decreased (*P* < 0.001).

### The relationship between the SIRI index and risk factors for cardiovascular disease

After performing coronary angiography, the participants were split into two groups: those with normal coronary arteries and those with coronary artery disease. Table 2 displays the results, which reveal notable differences between the CHD group and the normal group in terms of gender, age, SIRI index, TG, LPa, and GLU. These measures were greater in the CHD group (*P* < 0.001), indicating that males and older individuals were at a higher risk for CHD. The likelihood of CHD was also significantly increased in groups with higher SIRI index, TG, LPa, and GLU. More patients fell into the SIRI index quartile groups T2, T3, and T4 than the normal coronary artery group. In addition, the CHD group had significantly lower levels of HDL-C and ApoA1 (*P* < 0.001). Individuals in the CHD group also had a higher prevalence of hypertension, diabetes mellitus, and smoking compared to those in the normal coronary artery group (*P* < 0.001).

### Application of multifactorial logistic regression analysis of CHD by SIRI quartiles

Table 3 presents the results of the analysis, using patients with normal coronary arteries as the comparison group. The study revealed a significant association between a high prevalence of CHD and the fourth quartile of the SIRI index. Furthermore, the T4 group had a 2.25 times higher risk of CHD than the T1 group (OR ═ 2.25 [95% CI 1.73, 2.92]) (*P* < 0.05), even after accounting for various social, lifestyle, and disease characteristics, such as gender, age, smoking, hypertension, diabetes mellitus, and lipid levels.

**Table 2:** Clinical characteristics of CHD patients based on coronary angiography

**Characteristics**	**Normal coronary angiography (*n* ═ 1124)**	**Coronary lesion (*n* ═ 1866)**	***P***
Age (years)	57 (51, 66)	64 (56, 71%)	<0.001
Male	492 (43.8%)	1283 (68.8%)	<0.001
SIRI			<0.001
T1 (lowest)	394 (35.1%)	353 (18.9%)	
T2	321 (28.6%)	425 (22.8%)	
T3	260 (23.1%)	490 (26.3%)	
T4 (highest)	149 (13.3%)	598 (32.0%)	
TC (mmol/L)	4.55 (3.86, 5.26)	4.35 (3.56, 5.21)	<0.001
TG (mmol/L)	1.26 (0.93, 1.83)	1.34 (0.97, 2.02)	0.004
LDL-C (mmol/L)	2.80 (2.27, 3.28)	2.67 (2.10, 3.28)	<0.001
HDL-C (mmol/L)	1.20 (1.01, 1.41)	1.09 (0.92, 1.28)	<0.001
ApoA1 (g/L)	1.40 (1.22, 1.66)	1.32 (1.16, 1.56)	<0.001
ApoB (g/L)	0.87 (0.69, 1.05)	0.85 (0.66, 1.08)	0.652
Lpa (g/L)	0.100 (0.044, 0.247)	0.124 (0.051, 0.296)	0.001
PLT (10^9^/L)	204 (169, 242)	193 (158, 229)	<0.001
GLU (mmol/L)	4.94 (4.43, 5.65)	5.50 (4.72, 7.10)	<0.001
Smoking	330 (29.4%)	944 (50.6%)	<0.001
Hypertension	377 (33.5%)	935 (50.1%)	<0.001
Diabetes	81 (7.2%)	382 (20.5%)	<0.001

Data are given as median (IQR) or n (%). *P* < 0.001 were considered statistically significant. T1: The first SIRI index quartile; T4: The fourth SIRI index quartile; TC: Total cholesterol; TG: Triglyceride; LDL-C: Low-density lipoprotein; HDL-C: High-density lipoprotein; ApoA1: Apolipoprotein A1; ApoB: Apolipoprotein B; LPa: Lipoprotein a; PLT: platelet count; GLU: Glucose; CHD: Coronary heart disease.

**Table 3:** Multivariate logistic regression analysis of CHD by SIRI quartile

**Normal coronary angiography (*n* ═ 1124) Ref.CHD (*n* ═ 1866)**	**SIRI quartiles**			
	**T1 (lowest) OR (95% CI)**	**T2**	**T3**	**T4 (highest)**
Model 1	Ref.	1.47 (1.21, 1.81)^*^	2.10 (1.71, 2.59)^*^	4.48 (3.56, 5.64)^*^
Model 2	Ref.	1.23 (0.99, 1.53)	1.48 (1.18, 1.85)^*^	2.92 (2.28, 3.75)^*^
Model 3	Ref.	1.13 (0.90, 1.41)	1.29 (1.02, 1.64)^*^	2.25 (1.73, 2.92)^*^

Data are odds ratios (95% CI) of multivariate logistic regression. Model 1: Crude; Model 2: Sex and age adjustments; Model 3: Adjusted for model 2, smoking, hypertension, diabetes. *P < 0.05. GLU: Glucose; LDL-C: Low-density lipoprotein; TC: Total cholesterol; TG: Triglyceride; ApoA1: Apolipoprotein A1; LPa: Lipoprotein a; CHD: Coronary heart disease; OR: Odds ratios; CI: Confidence intervals.

### The relationship between the SIRI and the severity of coronary artery lesions

**Figure 2.**
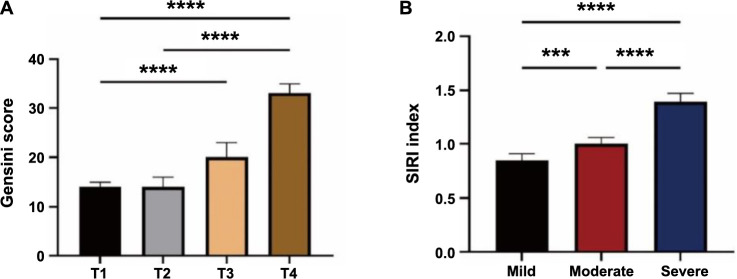
**Relationship between Gensini score and the SIRI in CHD patients**. (A) The Gensini score by SIRI index quartile; (B) The SIRI index by Gensini score tertile. ***P < 0.001, ****P < 0.0001. T1: The first SIRI index quartile; T4: The fourth SIRI index quartile; SIRI: Systemic Inflammatory Response Index; CHD: Coronary heart disease.

Patients with CHD were assessed using the Gensini score and the number of coronary artery branches with lesions. The patients were then divided into three groups (mild, moderate, and severe) based on their Gensini score tertiles (Table 4). The results of the study showed that the severity of coronary lesions varied significantly according to the SIRI index. In addition, in the group with multiple coronary lesions, the number of patients in the T4 group was significantly higher than in the other three groups. Gensini scores by quartiles of SIRI index and SIRI index by tertiles of Gensini index in CHD patients are shown in Figure 2. Gensini scores were significantly higher in the T4 group compared with the T1 and T2 groups (*P* < 0.001). The SIRI index was significantly higher in the severe coronary artery lesion group than in the mild and moderate coronary artery injury groups (*P* < 0.001). Poisson regression analysis between the SIRI index and Gensini score resulted in a significant positive effect of SIRI on Gensini score, and when the value of SIRI increased, the value of Gensini score also increased (*P* < 0.001) (Table 5).

**Table 4:** Association between the SIRI and coronary lesion severity in CHD patients

	**SIRI quartiles**				***P***
	**T1 (lowest) (*n* ═ 466)**	**T2 (*n* ═ 465)**	**T3 (*n* ═ 469)**	**T4 (highest) (*n* ═ 466)**	
Gensini score (tertiles)					<0.001
Mild (*n* ═ 574)	184	178	131	81	
Moderate (*n* ═ 657)	171	178	175	133	
Severe (*n* ═ 635)	111	109	163	252	
Coronary lesion branches					<0.001
Single vessel (*n* ═ 880)	259	252	206	163	
Multi-vessel (*n* ═ 986)	207	213	263	303	

*P* < 0.001 were considered statistically significant. CHD: Coronary heart disease; T1: The first SIRI index quartile; T4: The fourth SIRI index quartile; SIRI: Systemic Inflammatory Response Index.

**Table 5:** Poisson regression analysis of SIRI and Gensini score

**Variable**	**Estimate**	**St. Error**	***Z* value**	***P* value**	**RR (95% CI)**
Intercept	3.196	0.029	12571.369	<0.001	24.427 (23.100, 25.830)
SIRI	0.069	0.010	51.103	<0.001	1.071 (1.051, 1.092)

Dependent variable: Gensini score; SIRI: Systemic Inflammatory Response Index; St: Standard; RR: Respiratory rate.

### Comparison of SIRI and common leukocyte subtype ratio ROC curves and AUC

**Figure 3.**
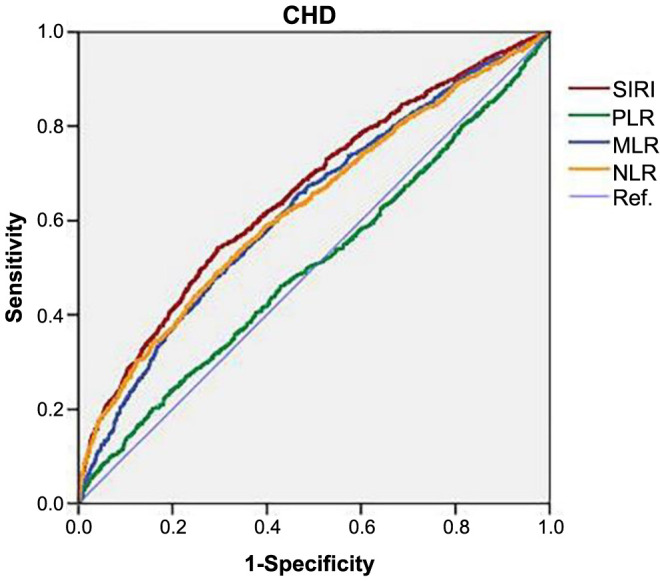
**ROC analyses of the SIRI.** CHD: Coronary heart disease; NLR: Neutrophil–lymphocyte ratio; PLR: Platelet–lymphocyte ratio; MLR: Monocyte–lymphocyte ratio; ROC: Receiver operating characteristic curve; SIRI: Systemic Inflammatory Response Index.

Table 6 shows the summarized results of ROC analysis of SIRI, platelet–lymphocyte ratio (PLR), monocyte–lymphocyte ratio (MLR), and neutrophil–lymphocyte ratio (NLR). The analysis revealed that SIRI had a larger AUC compared to PLR, MLR, and NLR, indicating its superior predictive power for CHD. The critical SIRI index for CHD was 0.247 (sensitivity: 54.3%; specificity: 70.4%) as depicted in Figure 3.

**Table 6:** ROC analyses of the SIRI

**Variable**	**AUC**	**95% CI**	***P***	**Sensitivity**	**Specificity**	**Youden’s index**
SIRI	0.657	0.638–0.677	<0.001	0.543	0.704	0.247
PLR	0.504	0.483–0.525	0.716	0.202	0.843	0.045
MLR	0.625	0.605–0.645	<0.001	0.655	0.535	0.190
NLR	0.628	0.608–0.648	<0.001	0.522	0.675	0.197

*P* < 0.001 were considered statistically significant. CHD: Coronary heart disease; PLR: Platelet–lymphocyte ratio; MLR: Monocyte–lymphocyte ratio; NLR: Neutrophil–lymphocyte ratio; CI: Confidence intervals; ROC: Receiver operating characteristic curve; AUC: Area under the curve; SIRI: Systemic Inflammatory Response Index.

## Discussion

The main pathophysiological mechanism of CHD is coronary AS. Recent studies have shown that AS is more than just lipid deposition; it is also an inflammatory disease [14], and that the onset, course, and outcomes of AS are significantly influenced by both local and systemic inflammatory responses [15, 16]. Currently, electrocardiograms, markers of myocardial injury, imaging signs, and other complete assessments are the most often utilized clinical approaches to identify CHD. Finding clinical indications is a straightforward process, and identifying inflammatory markers is useful in the diagnosis of CHD.

According to considerable literature, white blood cells, neutrophils [17], monocytes [18, 19], and lymphocytes [20–22] are inexpensive and readily available biomarkers of systemic inflammation, they are closely related to the occurrence of cardiovascular adverse events such as CHD [23, 24], stroke [25, 26], and all-cause mortality [27]. In addition to the classic inflammatory markers of blood cells, emerging composite metrics including NLR, MLR, and PLR are also gaining attention. The study found that the NLR was the strongest predictive marker for ACS and that it could be used as an adjunctive biomarker for the diagnosis of ACS. Furthermore, neutrophil and monocyte counts were significantly higher in ACS patients compared to healthy controls, and lymphocyte counts were significantly higher in controls compared to the ACS group [28]. Fan et al. [29] showed elevated levels of MLR and NLR were independent predictors of long-term MACE in patients treated with primary PCI for NSTEMI, and that the prognostic value for long-term MACE prediction was enhanced by combining these two inflammatory markers. In summary, numerous studies have been conducted on the calculation and analysis of NLR, PLR, and MLR as prognostic indicators [30–32]. Compared to white blood cell and subpopulation counts acquired directly from standard blood tests, they have a greater predictive value for the prediction of all-cause and cardiovascular mortality, as well as for the prognosis of disease [33, 34]. However, there has been no report on the relationship between these new indicators of inflammation and cardiovascular disease.

SIRI, a newly identified inflammatory marker, in combination with three blood markers, provides a more comprehensive and systematic assessment of the body’s inflammatory state. It has a strong correlation with systemic diseases and has been found to be associated with increased mortality and sepsis risk, as well as greater stroke severity. In comparison with other markers, such as NLR, PLR, LMR, and RDW, SIRI has shown superior predictive ability [35]. A retrospective study conducted at multiple centers utilized SIRI as a neonatal inflammatory marker in patients with rheumatoid arthritis (RA) to aid in diagnosis and assess disease activity. Results showed that SIRI had a larger area under the ROC curve compared to NLR, MLR, and PLR for predicting interstitial lung disease (ILD) associated with RA [36]. Elevated SIRI has also been linked to a higher risk of stroke, both subtypes, and all-cause mortality in a large prospective follow-up study. Additionally, it has been associated with an increased incidence of myocardial infarction in individuals over 60 years old [37]. Studies have demonstrated that SIRI is a strong and independent risk factor for adverse outcomes in patients with ACS undergoing percutaneous coronary intervention. Higher SIRI levels indicate more severe disease and improve the prognostic value of the GRACE risk score [38]. A large follow-up cohort study found that two novel inflammatory composite indices, SII and SIRI, were significantly associated with cardiovascular mortality and all-cause mortality. This highlights the importance of considering systemic inflammation for better prevention strategies [39]. Having shown that SIRI is also a strong independent predictor of MACE in patients with NSTEMI [40], that elevated levels of SIRI may reflect a dysregulation of the immune response characterized by an imbalance between pro- and anti-atherosclerotic immune networks, and that this imbalance leads to a transition from a stable to an unstable state of the plaque, which in turn leads to the occurrence of acute coronary events [41].

It is clear that SIRI is an increasingly utilized and non-invasive indicator of inflammation across various scientific disciplines. It is also a strong predictor of numerous inflammation-related disorders, including cardiovascular disease. In this study, we examined the correlation between SIRI and the risk of CHD. We observed a higher number of patients with coronary artery disease in the second, third, and fourth quartiles of SIRI compared to the group with normal coronary arteries. After adjusting for confounding factors, logistic regression analysis confirmed that the risk of coronary artery disease was significantly higher in the third and fourth quartiles of SIRI compared to the group with normal coronary arteries. The highest SIRI quartile was associated with a greater incidence of CHD compared to the lowest quartile (OR ═ 2.25 [95% CI 1.73, 2.92]) (*P* < 0.05). Furthermore, we evaluated the severity of coronary lesions in the afflicted population by using Gensini scores, and the Gensini score tertiles were used to represent mild, moderate, and severe coronary artery disease. SIRI in the third quantile of Gensini score was significantly higher than in the first and second quantiles, suggesting that patients with multi-vessel and severe coronary artery lesions typically had higher SIRI. When the value of the SIRI index grew, the value of the Gensini score similarly increased (*P* < 0.05) in the Poisson regression analysis. The SIRI index had a bigger area under the ROC curve (AUC ═ 0.657, *P* < 0.05) than the inflammation indices NLR, MLR, and PLR, which were based on the combination of two blood indicators. As a result, the SIRI index was more valuable for identifying CHD.

At present, systemic inflammation is largely studied for its prognostic value in cardiovascular events (MACE). However, few studies have examined the diagnostic significance of inflammation-related markers in cardiovascular disease. In the early stages of the disease, some indicators of heart damage and the electrocardiogram may not reveal abnormalities. The diagnostic gold standard for CHD is coronary angiography, an invasive and costly procedure that is not feasible in rural or under-resourced areas. Furthermore, some patients with no symptoms or existing CHD may not be willing to undergo this invasive procedure. Therefore, our study aimed to assess the correlation between SIRI and the diagnosis of coronary artery disease. The analysis of the results indicated that SIRI can serve as an independent predictor of the development of coronary artery disease and be used as a complementary diagnostic index for this condition.

One noteworthy protein found in human plasma is albumin, which is commonly used as an indicator of nutritional status [42]. Chronic inflammation can negatively impact its production. Studies have demonstrated that serum albumin levels can predict long-term mortality in patients with implanted dual-chamber permanent pacemakers [43]. Albumin is an important component of the Naples score (NS), a tool used to predict adverse cardiovascular events, which can also be used to assess long-term mortality risk in patients with ST-elevation myocardial infarction (STEMI) undergoing primary percutaneous coronary intervention [44]. The Naples prognostic score (NPS) may have the potential to predict long-term mortality in patients with acute pulmonary embolism (APE) [45]. Albumin in the development and progression of CHD is a topic that deserves to be explored in depth in future studies.

## Limitations

There are several limitations to this study. First, the results may be influenced by recall bias since it was a retrospective observational study. Second, our findings were limited due to the study being conducted at a single center. Hence, to improve the accuracy and generalizability of SIRI, future multicenter studies with more extensive experiments on this topic are necessary.

## Conclusion

Despite various traditional influencing factors, the SIRI index was found to have a positive correlation with AS. In fact, higher SIRI values were significantly associated with the severity of the disease. The findings of this study suggest that SIRI can effectively identify high-risk patients with coronary artery disease, making early intervention crucial for their prognosis. The emergence of new inflammatory markers in cardiovascular disease is expected to be a prominent area of research in the future.
